# Somatic SMARCB1 Mutation in Sporadic Multiple Meningiomas: Case Report

**DOI:** 10.3389/fneur.2018.00919

**Published:** 2018-10-26

**Authors:** Alice S. Wang, Ali O. Jamshidi, Nathan Oh, Ronald Sahyouni, Behdokht Nowroozizadeh, Ronald Kim, Frank P. K. Hsu, Daniela Bota

**Affiliations:** ^1^Department of Neurological Surgery, University of California-Irvine Medical Center, Orange, CA, United States; ^2^Department of Pathology and Laboratory Medicine, University of California-Irvine Medical Center, Orange, CA, United States; ^3^Chao Comprehensive Cancer Center, University of California-Irvine Medical Center, Orange, CA, United States; ^4^Department of Neurology, University of California-Irvine Medical Center, Orange, CA, United States

**Keywords:** sporadic multiple meningiomas, familial multiple meningiomas, SMARCB1, NF2, somatic mutation, germline mutation

## Abstract

**Background:** Multiple intracranial meningiomas account for <10% of all meningiomas. Familial multiple meningiomas have been linked to germline mutations in two genes: *neurofibromatosis type 2* (NF2) and *SWIch/Sucrose Non-Fermentable (SWI/SNF)-related matrix-associated actin-dependent regulator of chromatin subfamily B member 1* (SMARCB1). Sporadic multiple meningiomas have been associated with somatic NF2 mutations and, to date, there has been no case related to somatic SMARCB1 mutations. Here, we describe the first case.

**Case Report:** A 45-year-old female suffered a head trauma while snowboarding. Subsequent to her injury, she experienced persistent headache, nausea, vomiting, dizziness, and flashing lights in the right eye. Magnetic resonance imaging (MRI) of her brain revealed multiple intracranial meningiomas. She underwent a two-staged craniotomy to remove frontal/parietal/temporal and occipital extra-axial tumors. Pathology confirmed the masses as meningiomas, WHO Grade I. Tumor genetic testing was positive for SMARCB1 mutation but blood genetic testing was negative for SMARCB1 mutation.

**Conclusion:** In sporadic multiple meningiomas, somatic NF2 mutations are usually the suspected genetic alternations. Our case illustrates that somatic SMARCB1 mutation is another genetic risk factor for sporadic multiple meningiomas, albeit rare.

## Introduction

The phenomenon of multiple meningiomas is rare, accounting for < 10% of all meningiomas (1). Multiple meningiomas can be categorized as familial or sporadic. Familial multiple meningiomas can manifest due to neurofibromatosis type 2 (NF2) disease, in which the NF2 gene on chromosome 22 mutates the growth inhibitory function of Merlin. Alternatively, familial multiple meningiomas can be inherited in an autosomal dominant fashion without the involvement of the NF2 gene (2). Sporadic multiple meningiomas are usually associated with somatic NF2 mutations (heinrich). Somatic SMARCB1 mutations have been associated with sporadic meningiomas in the literature; however, these were sporadic *solitary* meningiomas (3–5). To date, there is no documented report of somatic SMARCB1 mutation as the underlying genetic alternation for sporadic *multiple* meningiomas. The authors describe the first case of a somatic SMARCB1 mutation in a patient with sporadic multiple meningiomas who harbors no NF2 mutations.

## Case report

**Figure 1:**
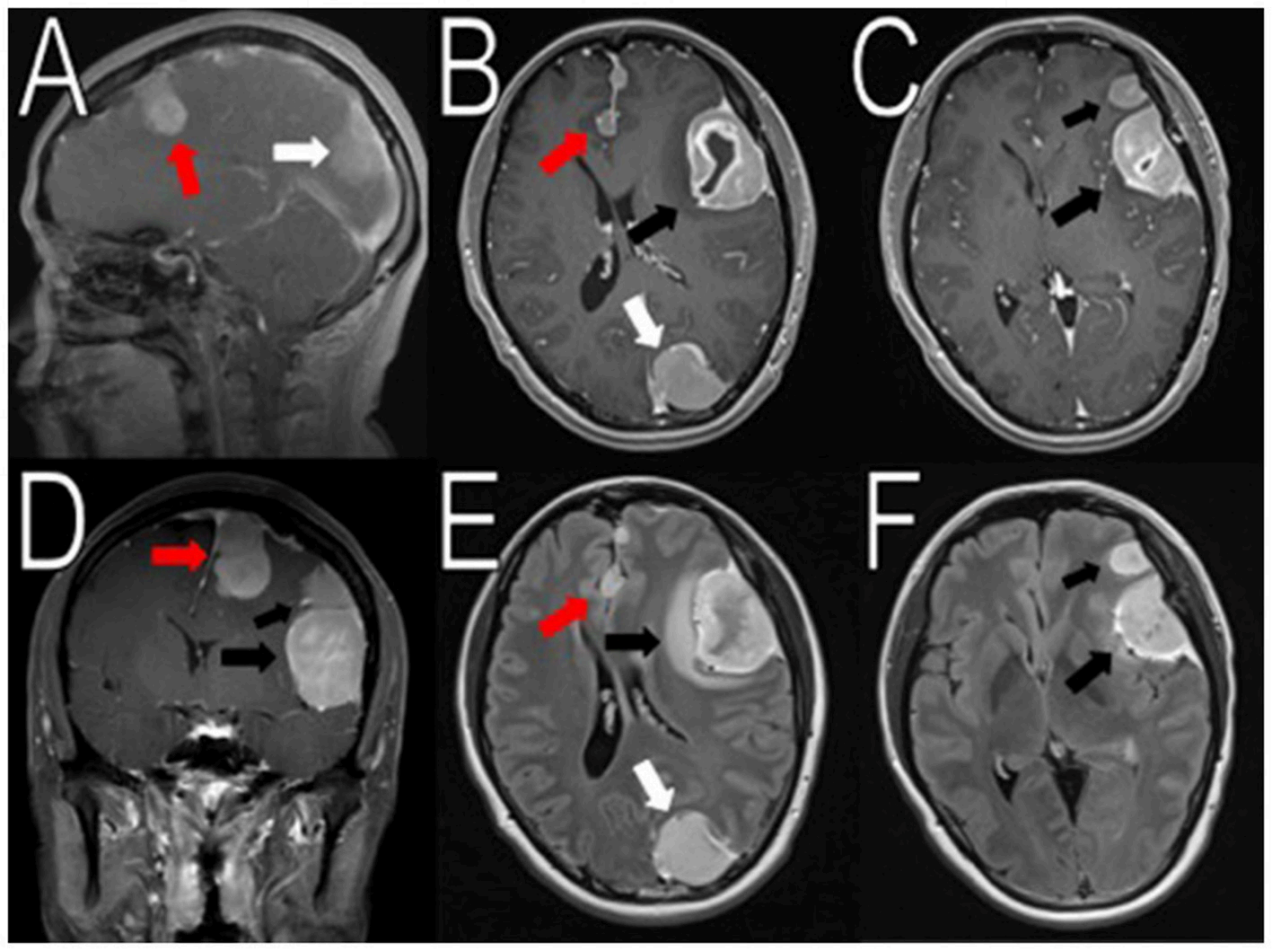
Initial imaging showing multiple menigniomas. Preoperative contrasted T1 and T2 MRI scans reveal multiple meningiomas. The left calcified parafalcine lobulated mass (2 × 2.2 × 3.3 cm, AP, TV, CC) was associated with vasogenic edema (red arrows in **A,B, D–E**). In the left frontal temporal convexity, there was a 4.5 × 2.9 × 4.1 cm (AP, TV, CC) mass with another 2.3 × 2.0 × 1.9 cm (AP, TV, CC) mass located superior to it (black arrows in **B–F**). In the occipital lobe, the mass was measured to be 2.6 × 2.9 × 3.9 cm (AP, TV, CC) (white arrows in **A,B,E**). There was an 8 mm rightward midline shift. The images seemed to suggest neurofibromatosis type 2.

The authors report the case of a 45-year-old female who suffered a snowboarding accident and presented several days following the event complaining of persistent headache, nausea, vomiting, dizziness, and photic auras in the right eye. She visited an urgent care facility twice and during these visits, no imaging was obtained. A neurologist saw her 18 days after the accident. An MRI scan was ordered that showed multiple masses some of which harbored hemorrhagic components: a left frontal parafalcine, calcified 2.0 × 2.2 × 3.3 cm [anteroposterior (AP), transverse (TV), craniocaudal (CC)] mass with associated vasogenic edema, a 4.5 × 2.9 × 4.1 cm (AP, TV, CC) mass in the left frontotemporal convexity with another mass measuring approximately 2.3 × 2.0 × 1.9 cm (AP, TV, CC) located just superiorly, and a 2.6 × 2.9 × 3.9 cm (AP, TV, CC) mass in the left occipital lobe (Figure 1). Additionally, there was an 8 mm left to right midline shift (Figure 1). Her findings were most consistent with multiple meningiomas. There was also a possible vestibular schwannoma measuring 0.7 × 1.4 × 0.7 cm (AP, TV, CC) in the left internal auditory canal (figure not shown). Initially, she was thought to have NF2. Pre-surgical tumor embolization and a two-staged surgery were recommended. The patient had successful embolization of the left middle meningeal artery and left posterior meningeal artery.

**Figure 2:**
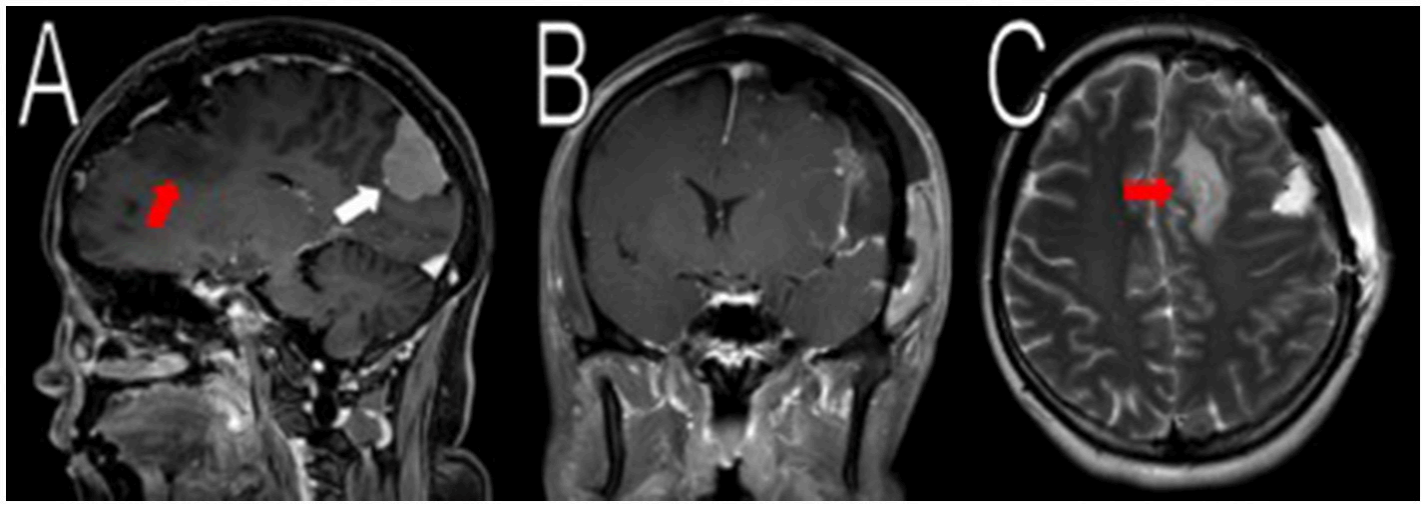
Resection of the frontal/parietal/temporal mass. Post-operative MRI contrasted T1 and T2 scans showed resection of the meningiomas in the left frontal/parietal/temporal convexity with expected post-operative changes (red arrows in **A,C**, not shown in **B**). The occipital lobe mass was visible from the sagittal view (white arrow in **A**).

**Figure 3:**
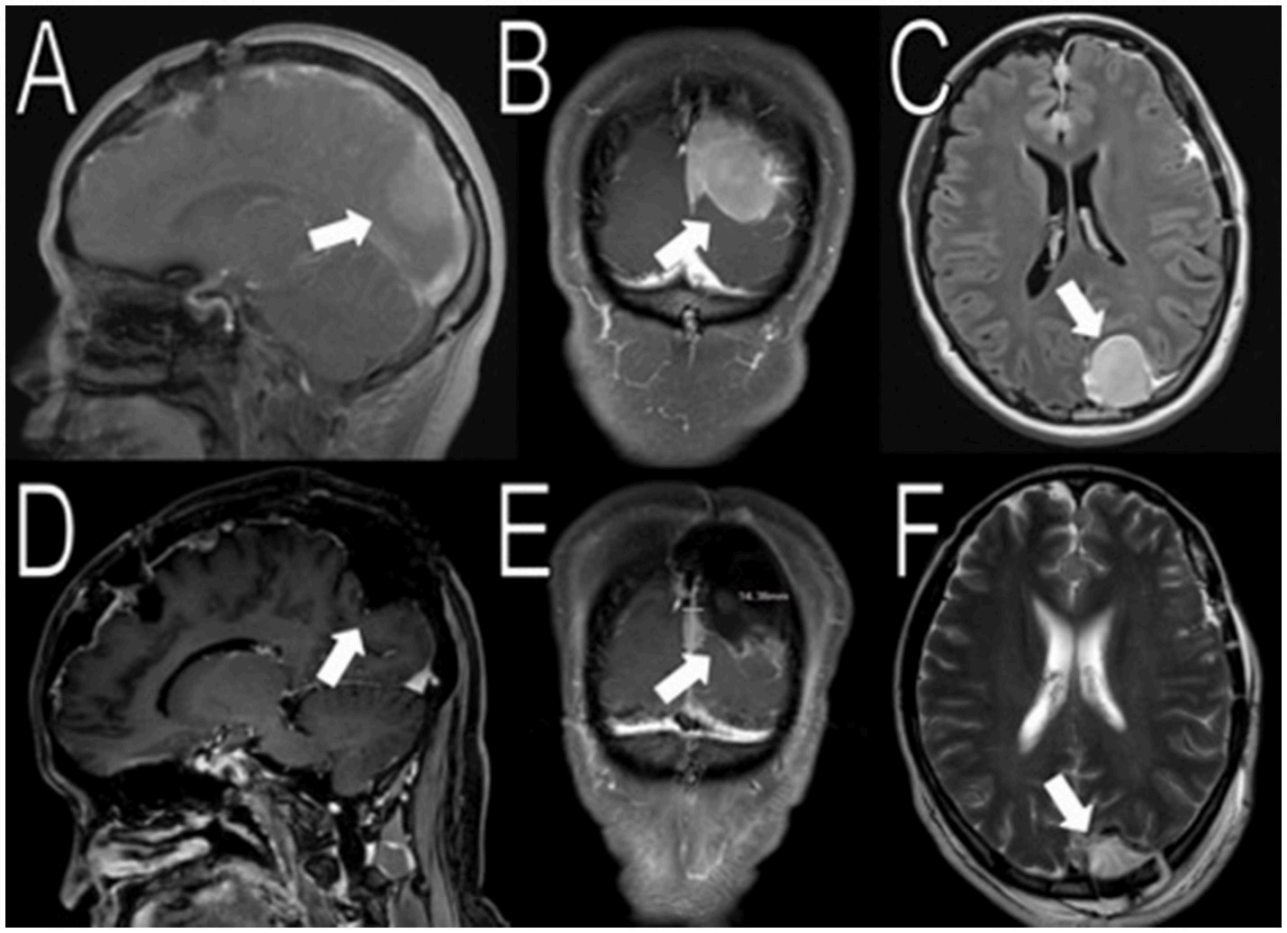
Resection of the occipital mass. Preoperative MRI showed a 2.6 × 2.9 × 3.9 cm (AP, TV, CC) mass in the left occipital convexity (white arrows in **A–C**). Immediate post-operative MRI showed removal of the mass (white arrows in **D–F**).

**Figure 4:**
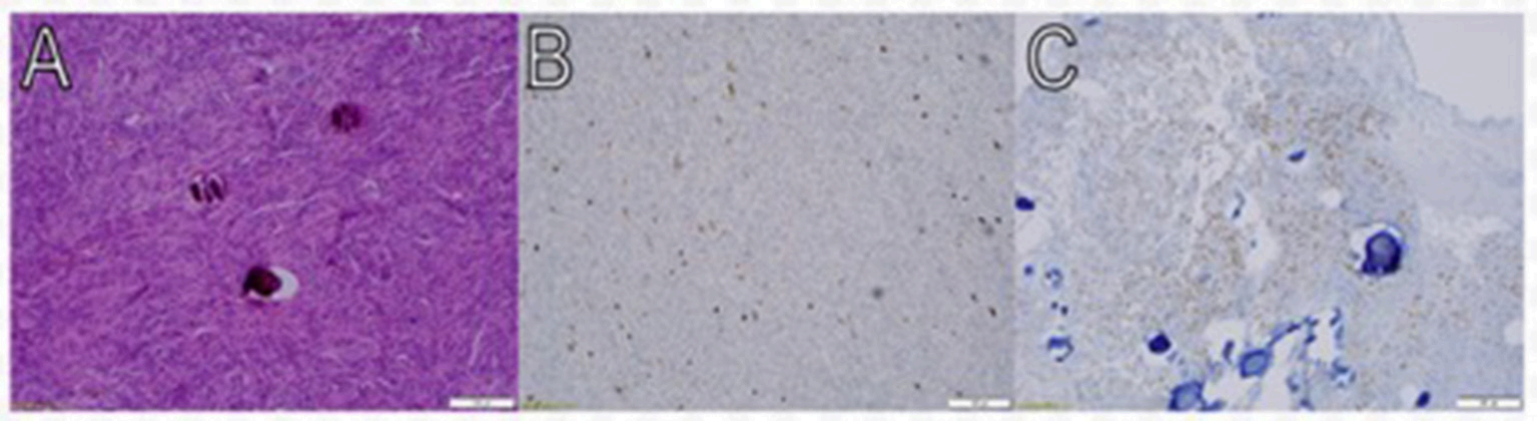
Pathologic findings of meningothelial meningioma. Macroscopic view at low power magnification (10X) shows meningothelial cells that are packed together in fascicles and whorls in a syncytial pattern. The nuclei are round and uniform, and occasional psammoma bodies are noted **(A)**. Approximately 2–3% of tumor cells nuclei are immunoreactive for Ki- 67 (Immunostainx100) **(B)**. Some of the tumor cells are immunoreactive for PR (Immunostainx100) **(C)**.

**Figure 5:**
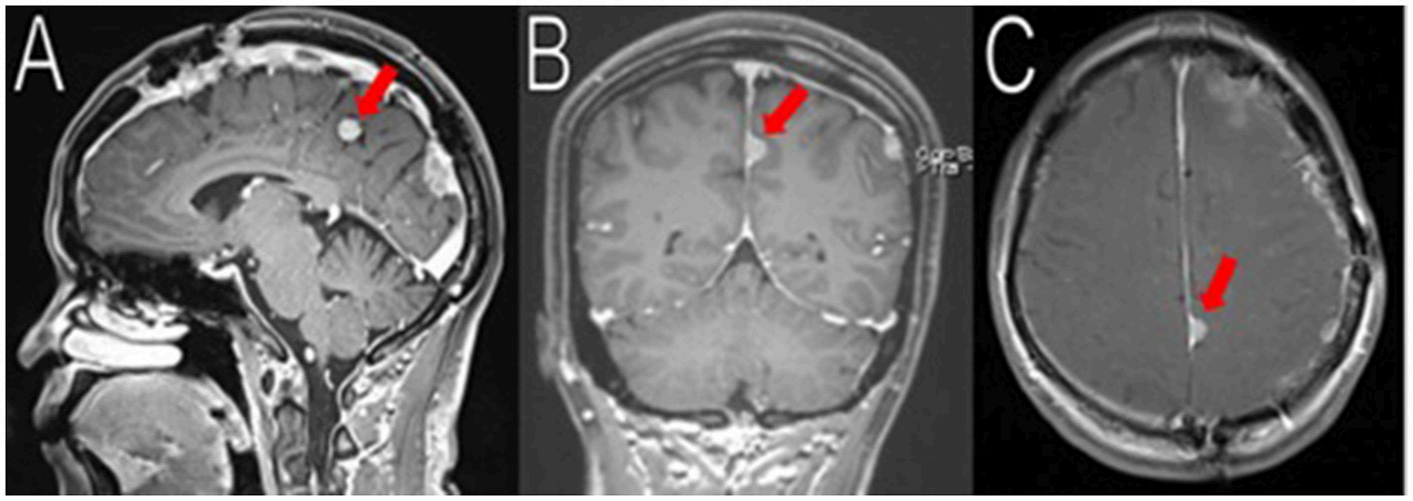
Latest imaging. Her most recent MRIs (16 months after her last surgery) show multiple enhancing extra-axial masses, stable compared to her immediate post-operative MRIs. Here is a stable 1.8 cm (superior-inferior) meningioma arising from the left posterior falx, adjacent to the prior resection cavity (red arrows in **A–C**). No recurrence observed.

The first surgical stage involved a left-sided craniotomy for resection of the frontal-parietal-temporal meningiomas; pathology reported WHO Grade I meningiomas with a low/moderate proliferation index (percentages of positive Ki-67 tumor nuclei: left occipital mass: 2–3%; midline frontal mass: 3–4%; and left frontal mass: 1–2%; Figure 1). NF-2 blood testing (NEUROFIBROMATOSIS TYPE 2 SEQUENCING AND DELETION/DUPLICATION ANALYSIS IN Blood, UAB), which has a mutation detection rate in leukocytes of 93% was negative. This specific study detects truncating mutations (nonsense, frameshift, splicing mutations including deep intronic splice mutations), missense mutations, multi-exon deletions or duplications, and total gene deletions. Post-operative MRIs showed resection of the meningiomas in the left frontal/parietal/temporal convexity (Figure 2). Seven months after her first-stage surgery, she underwent the second surgical stage: left occipital craniotomy for resection of a 2.9 × 2.7 × 4.2 cm (AP, TV, CC) mass (Figure 3). A specimen from the left occipital mass was sent to pathology which classified it as meningioma, WHO Grade I with a Ki-67 of 2–3% (Figure 4). Her most recent post-operative MRI scans at 16 months of follow up show multiple stable enhancing extra-axial masses compared to her immediate post-operative ones with no new lesions observed (Figure 5).

**Figure 6:**
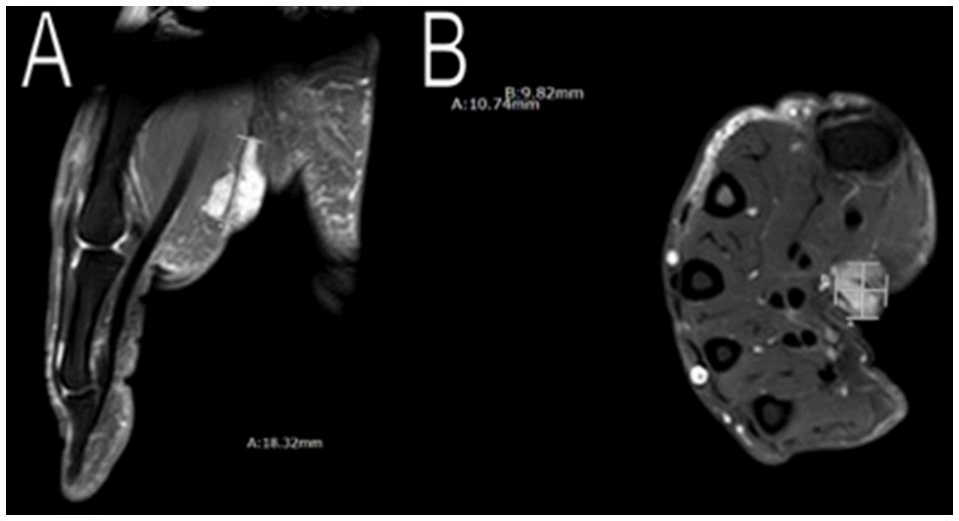
Imaging of her right palm mass. MRI scans showed an approximately 11 × 10 × 20 mm mass in her right palm. Plastic surgery excised the mass and pathology confirmed a benign nerve sheath tumor, consistent with Schwannoma **(A,B)**.

In addition to having multiple meningiomas, she also had a soft tissue mass in her right palm and moles in her left axilla. MRI scans showed the mass to be approximately 11 × 10 × 20 mm (Figure 6) with unclear pathology, and the patient was referred to plastic surgery. The patient underwent surgery to excise her cystic mass and pathology found it to be a benign nerve sheath tumor, consistent with Schwannoma with positive immunostain S-100. As for the moles on her left armpit, a shaved biopsy was obtained and pathology confirmed pigmented seborrheic keratosis, consistent with an atypical mole.

Because she was NF2 negative, further genetic testing was pursued for both somatic and germline mutations. Foundation Medicine, Inc. (Cambridge, Massachusetts, USA) tested her tumor tissue positive for SMARCB1 mutation (but not for NF2) while her blood genetic testing was negative for SMARCB1 mutation.

## Discussion

Multiple meningiomas occur in < 10% of patients with meningiomas (1). Multiple meningiomas, whether sporadic or familial, seem to have a clonal origin, rather than an isolated formation of unrelated tumors (6, 7). Cerebrospinal fluid may be the vehicle of transportation for spreading the clonal tumor cells to various locations in the central nervous system (7).

Neurofibromatosis type 2 disease harbors mutations in the NF2 gene, which are well-known risk factors associated with familial multiple meningiomas and sporadic multiple meningiomas (3, 4, 8). The NF2 gene is located at chromosome 22q12.2 and regulates the production of Merlin (schwannomin), a tumor suppressor protein. Approximately 50% of patients with NF2 disease inherit germline NF2 mutations and develop multiple meningiomas (9). The type and position of mutations in the NF2 gene contribute to a differential risk of developing multiple meningiomas. For example, truncating mutations at the proximal 5′ end of the gene pose a higher risk than non-truncating mutations at proximal 3′ end of the gene (10). The most common genetic risk factor is somatic NF2 mutations in sporadic multiple meningiomas (3–5).

Another gene of particular interest is SMARCB1 (aka INI1 and SNF5), which is located on chromosome 22 but at 22q11.23 (4). SMARCB1 is a tumor suppressor protein that is part of the SWI/SNF complex that remodels chromatin structures for transcription (11). For an extensive discussion of the underlying pathophysiological mechanisms, we refer the reader to Kalimuthu et al. (12). Mutations in the SMARCB1 gene have been associated with malignant rhabdoid tumor, schwannomatosis, and meningiomas (11, 13, 14). Bacci et al. found in a family study that germline SMARCB1 mutations, with no somatic SMARCB1 or NF2 mutations, are associated with familial schwannomatosis and multiple meningiomas (14). In a follow-up study by Hadfield et al., they found no germline SMARCB1 mutations in multiple meningiomas. This study contained both sporadic and familial multiple meningiomas (5:1 ratio in patient proportion); therefore, these sample differences could have contributed to this finding. Additionally, the sample size was small; only 6/47 patients had tumor DNA and blood DNA available for analysis (15). In another family study done by Christiaans et al., germline SMARCB1 mutation and somatic NF2 mutations were found in familial multiple meningiomas. They proposed the four-hit mechanism involving both tumor suppressor genes SMARCB1 and NF2 (13). Interestingly, van den Munckhof et al. found that germline SMARCB1 mutation and somatic NF2 mutations preferentially localized the cranial meningiomas at the falx cerebri (16).

Somatic SMARCB1 mutations have also been studied in meningiomas. Somatic SMARCB1 mutations have been reported in sporadic meningiomas; however, a closer examination of these reports reveals the meningiomas were *solitary* (17, 18). To date, there is no report of somatic SMARCB1 mutations in sporadic *multiple* meningiomas. The authors describe the first case in which somatic SMARCB1 mutation contribute to the development of sporadic multiple meningiomas.

Initially, the authors hypothesized that the patient likely had a founder germline NF2 mutation given that she has no family history of NF2. Once it was determined that she had no NF2 mutations in either blood or the tumor tissue, the authors tested for other mutations. Fountain Medicine, Inc. (Cambridge, Massachusetts, USA) found a mutation in the SMARCB1 gene. Her blood test was negative for SMARCB1 mutation, indicating that she did not harbor known or expected germline SMARCB1 mutations. It is unlikely, although still plausible, that the genetic testing yielded a false negative result, however, based on the family history and her clinical presentation, this is less likely. Therefore, she had a somatic mutation in the SMARCB1 gene. Because she harbored only this mutation and she developed sporadic multiple meningiomas, the authors believe somatic SMARCB1 mutation poses a genetic risk for sporadic multiple meningiomas.

## Conclusion

Sporadic multiple meningiomas are rare phenomena and germline or somatic NF2 mutations are usually the culprit. Here, the authors describe the first case in which somatic SMARCB1 mutation is responsible for the development of sporadic multiple meningiomas. Somatic SMARCB1 mutation is a genetic risk factor for sporadic multiple meningiomas and should be considered for testing when markers for NF2 are negative in similar clinical situations as it could be a marker for possible future therapeutics.

## Ethics statement

This study received an exemption from our institutional review board due to the nature of the study and the fact that it is a case report with fewer than 3 subjects.

## Consent

Written, informed consent was obtained from the participant for the publication of this case report.

## Author contributions

AW, AJ, NO, RS, BN, RK, FH, and DB contributed to the design and implementation of the research, to the analysis of the results and to the writing of the manuscript.

### Conflict of interest statement

The authors declare that the research was conducted in the absence of any commercial or financial relationships that could be construed as a potential conflict of interest.

## Abbreviations

SWI/SNF: SWItch/Sucrose Non-Fermentable
SMARCB1: SWI/SNF-related matrix-associated actin-dependent regulator of chromatin subfamily B member 1
NF2: neurofibromatosis type 2
MRI: magnetic resonance imaging
WHO: World Health Organization
AP: anteroposterior
TV: transverse
CC: craniocaudal
q: long arm of the chromosome.
